# Letter from the Editor-in-Chief

**DOI:** 10.19102/icrm.2018.090106

**Published:** 2018-01-15

**Authors:** Moussa Mansour


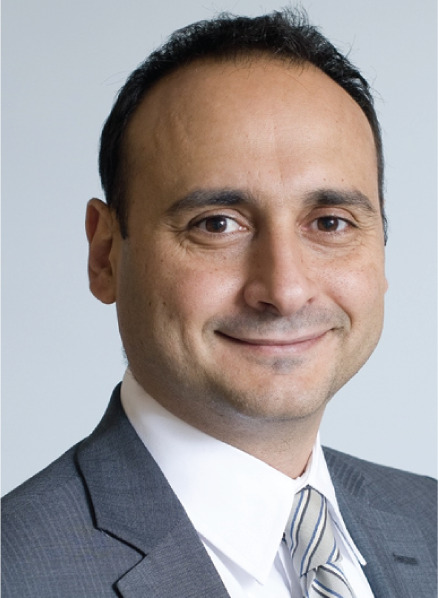


Dear Readers,

This issue of *The Journal of Innovations in Cardiac Rhythm Management* contains an important article by Sanchez et al. titled “Optimal Ablation Techniques for Ventricular Tachycardia Management.” In it, the authors discuss the mechanism of ventricular tachycardia (VT) and review some of the ablation strategies that are currently being used.

The number of ablations performed for VT has significantly grown in the last few years. Patients with all sorts of cardiomyopathies are living longer because of the advances that have been made in the treatment of congestive heart failure and coronary artery disease and the widespread use of ventricular assist and implantable cardiac rhythm management devices. Many of these patients develop VT that is often resistant to pharmacological therapy, thus necessitating ablation instead. Indeed, some studies have projected that the number of VT ablations will double over the next seven years.

Despite the increase in the number of VT ablations, however, this procedure remains challenging. The success rate for this arrhythmia is less than that for other arrhythmias and the complication rate is higher. One reason for these differences is that patients undergoing VT ablation are generally sicker than those undergoing ablation for other arrhythmias. However, I believe the most important reason for this difference is that the technology for VT ablation is not developing at the same rate as the technologies for other arrhythmias such atrial fibrillation (AF). In fact, most tools used in the ablation of VT were initially developed and tested for AF ablation. While the creation of transmural lesions is not hard to achieve in the atrium, it remains a major problem in the ventricle. Thus, there is a critical need to develop tools that are dedicated for the mapping and ablation of VT.

In addition to the limitations in technology, the evidence-based science in VT ablation is also lagging. The field of VT ablation is limited by many unresolved controversies and the optimal technique for VT ablation remains to be determined. Large clinical studies conducted routinely in AF ablation are lacking in data on VT ablation. Thus, there is a serious need to design clinical trials to test different ablation strategies in order to provide guidance for the ablation of VT.

I hope that you enjoy reading this issue of the journal. Best wishes for a happy and healthy new year.

Sincerely,


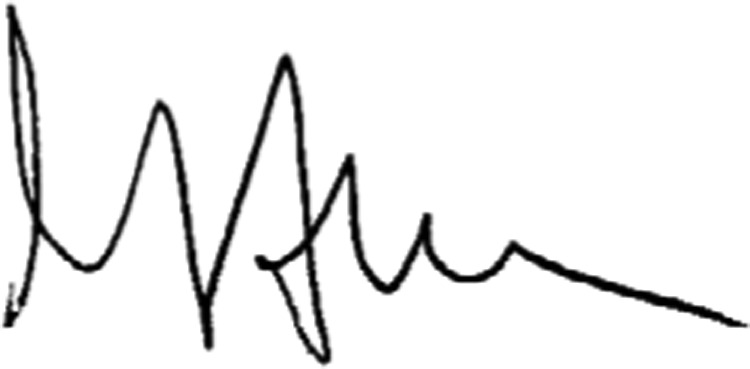


Moussa Mansour, md, fhrs, facc

Editor-in-Chief

The Journal of Innovations in Cardiac Rhythm Management

MMansour@InnovationsInCRM.com

Director, Atrial Fibrillation Program

Jeremy Ruskin and Dan Starks Endowed Chair in Cardiology

Massachusetts General Hospital

Boston, MA 02114

